# Recommendations for Genetic Variation Data Capture in Developing Countries to Ensure a Comprehensive Worldwide Data Collection

**DOI:** 10.1002/humu.21397

**Published:** 2010-11-18

**Authors:** George P Patrinos, Jumana Al Aama, Aida Al Aqeel, Fahd Al-Mulla, Joseph Borg, Andrew Devereux, Alex E Felice, Finlay Macrae, Makia J Marafie, Michael B Petersen, Ming Qi, Rajkumar S Ramesar, Joel Zlotogora, Richard GH Cotton

**Affiliations:** 1 University of Patras, School of Health Sciences, Department of PharmacyPatras, Greece; 2 Department of Genetic Medicine, Faculty of Medicine and Princess Al-Jawhara Center of Excellence in Research of Hereditary Disorders, King Abdulaziz UniversityJeddah, Saudi Arabia; 3 Department of Pediatrics, Riyadh Military Hospital and Stem Cell Therapy Program, King Faisal Specialist Hospital and Research CenterRiyadh, Saudi Arabia; 4 Molecular Pathology Unit, Kuwait UniversityKuwait; 5 University of Malta, Faculty of Health Sciences, Department of Applied Biomedical SciencesMsida, Malta; 6 NGRL Manchester, Department of Medical Genetics, St. Mary's HospitalManchester, United Kingdom; 7 University of Malta, Department of Physiology and Biochemistry, Laboratory of Molecular GeneticsMsida, Malta; 8 Department of Colorectal Medicine and Genetics and University of Melbourne, The Royal Melbourne HospitalAustralia; 9 Clinical Genetics, Kuwait Medical Genetics Centre, Maternity Hospital, Sabah Medical AreaKuwait; 10 Institute of Child Health, Department of GeneticsAthens, Greece; 11 Center for Genetic and Genomic Medicine, The First Affiliated Hospital of Zhejiang University School of Medicine and James Watson Institute of Genomic Sciences, People's Republic of ChinaSouth Africa; 12 MRC Human Genetics Research Unit, Division of Human Genetics, Institute for Infectious Diseases and Molecular Medicine, Faculty of Health Sciences, University of Cape TownSouth Africa; 13 Department of Community Genetics, Public Health Services, Ministry of HealthJerusalem, Israel; 14 Genomic Disorders Research Centre, Howard Florey Institute, Melbourne, Australia & Faculty of Medicine, Dentistry and Health Sciences, The University of MelbourneAustralia

**Keywords:** developing countries, national/ethnic mutation databases, populations, genetic variation

## Abstract

Developing countries have significantly contributed to the elucidation of the genetic basis of both common and rare disorders, providing an invaluable resource of cases due to large family sizes, consanguinity, and potential founder effects. Moreover, the recognized depth of genomic variation in indigenous African populations, reflecting the ancient origins of humanity on the African continent, and the effect of selection pressures on the genome, will be valuable in understanding the range of both pathological and nonpathological variations. The involvement of these populations in accurately documenting the extant genetic heterogeneity is more than essential. Developing nations are regarded as key contributors to the Human Variome Project (HVP; http://www.humanvariomeproject.org), a major effort to systematically collect mutations that contribute to or cause human disease and create a cyber infrastructure to tie databases together. However, biomedical research has not been the primary focus in these countries even though such activities are likely to produce economic and health benefits for all. Here, we propose several recommendations and guidelines to facilitate participation of developing countries in genetic variation data documentation, ensuring an accurate and comprehensive worldwide data collection. We also summarize a few well-coordinated genetic data collection initiatives that would serve as paradigms for similar projects. Hum Mutat 31:1–8, 2010. © 2010 Wiley-Liss, Inc.

## Introduction

The recent elucidation of the genome sequence of several individuals, such as Causacians (Jim Watson [Wheeler et al., 2008] and Craig Venter [Levy et al., 2007]), West African [Bentley et al., 2008], and Han Chinese [Wang et al., 2008], suggests that a large number of single nucleotide polymorphisms (SNPs) and other sequence variation exists in the human population. Estimates from African genetic diversity and the Pan Asian SNP initiative suggest that approximately 80–90% of human genomic variation resides in the world's developing countries [Hinds et al., 2005]. It is therefore obvious that there is a huge need to comprehensively document the extant genetic variation in various ethnic groups and populations around the globe to ensure maximal data coverage.

Since 2002, there have been several initiatives to thoroughly document the genetic heterogeneity in various populations worldwide in National/Ethnic Mutation databases (NEMDBs), a large number of which reside in developing countries. Despite the fact that data content is rather homogeneous, facilitated by the existence of an off-the-shelf database management system [van Baal et al., 2010], these databases have been developed without appropriate guidelines and recommendations to ensure not only a thorough description of common and rare genetic diseases in these populations but also maximal visibility and usefulness of these resources in the respective countries while maintaining the individual local ethical and cultural values in them. Furthermore, developing countries are often a valuable resource of clinical samples that derive from consanguineous families with very unusual phenotypes. These genotype and phenotype data are worth being recorded in NEMDBs not only to make them readily available to interested parties but also to develop and implement genetic epidemiological approaches for estimating the relative frequency of rare autosomal recessive disorders and an estimate of their prevalence in these populations.

Here, we propose a number of guidelines and recommendations that would assist in genetic variation data capture in developing countries to ensure a comprehensive worldwide data collection, one of the aims and goals of the Human Variome Project (HVP) [Cotton et al., 2007; Kaput et al., 2009] (http://www.humanvariomeproject.org). These guidelines are largely the result of discussions among the participants of the developing countries working group during the International HVP Planning and Implementation meetings in May 2008 and 2010, respectively, coming from a wide range of developed and developing countries and extended, based on ongoing data documentation efforts in these countries.

## Background

Any formal attempt to identify the extent of genetic variation must include geographical regions that have not been included in previous haplotype mapping projects, for example, the HapMap project, particularly because the latter project included racial and not population groups. Although the Population Reference Sample (POPRES) will address some of these missing populations [Nelson et al., 2008], this effort is again designed as a mapping project that is not expected to focus on functional polymorphisms or mutations. Also, the Arab region has long played a key role in discovering new genes and it is really awkward why this region has not been so actively involved in today's genomics research and genome-wide association studies.

Hence, one of the main activities of the HVP is the analysis and subsequent inclusion of genetic variation data from clinical samples originating from diverse populations or ethnic groups [Cotton et al., 2009; Kaput et al., 2009]. The distinct advantage of some ethnic populations is the opportunity to study genetic diseases due to: (1) large family size, (2) consanguinity, and (3) potential founder effects [Saadallah and Rashed, 2007]. In fact, several disorders have been described for the first time in certain populations residing in developing countries, for example, Sanjad-Sakatti syndrome, Teebi-Shaltoot syndrome, Teebi Hypertelorism, and others. Based on the abovementioned advantages, developing nations are regarded as major contributors to the HVP even though biomedical research has not been the focus of these countries, despite such activities being likely to produce economic and health benefits for all [Kaput et al., 2009; Singer and Daar, 2001].

The NEMDBs are one of the main components of the HVP that could accommodate the extant genetic heterogeneity in the developing countries and, for that matter, all countries. In particular, the NEMDBs are mutation depositories needing continuous updating, recording extensive information over the described genetic heterogeneity of an ethnic group or population (reviewed in [Patrinos, 2006]). NEMDBs can help optimize national molecular diagnostic services, by stratifying the corresponding mutation spectra in various inherited diseases, and assist in interpreting diagnostic test results in countries with heterogeneous populations, particularly in minority ethnic groups. NEMDBs can also contribute toward the elucidation of the origin and migration of populations, acting as platforms for comparative genomic studies that can reciprocally provide insights into, for example, the demographic history of human populations, patterns of their migration and admixture, gene/mutation flow, etc. [Patrinos, 2006]. Such comprehensive databases would include information on genotype and reasonable phenotype, in a level of detail that should be agreed by the community, which would also enable those interested in these diseases to investigate the possible influence of geographical environment or genetic modifiers on expression of the disease/phenotype. Examples include the genetics of obesity, hypertension, or type II diabetes. Relevant genotype/phenotype collection projects, such as GEN2PHEN (http://www.gen2phen.org) or P3G (http://www.p3g.org) could serve as models to establish the necessary requirements for the depth of the recorded genotype and phenotype information.

## Recommendations

The recommendations and guidelines that follow (summarized in Box 1) are intended to facilitate genetic variation data capture in developing countries, either by suggesting specific measures aiming to enhance awareness among clinicians, bioscientists, and the general public about the range of commonest genetic disorders suffered by certain populations and/or ethnic groups, or by sharing the experience from ongoing projects to document genetic heterogeneity in different countries.


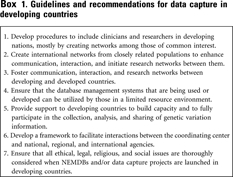


### 1. Develop Procedures to Include Clinicians and Researchers in Developing Nations, Mostly by Creating Networks Among Those of Common Interest

This would be particularly important for clinicians/researchers working in underresourced institutes and research environments. Where construction of a NEMDB is decided, selected representatives from participating laboratories, mainly established scientists and principal investigators involved in human genetics, should take the lead in this effort. These individuals would form the advisory committee of the NEMDB with one of them assigned as national coordinator with a defined (e.g., yearly) term. These members will then assign eligible members from their groups that would serve as data curators responsible for data entry/modification.

Such efforts would flourish if placed under the umbrella of specific scientific initiatives or consortia (see also below), such as the Human Genome Variation Society (HGVS; http://www.hgvs.org), a Society that generates recommendations and guidelines toward human genome variation capture, the HVP, and ORPHANET (http://www.orpha.net), a database of information on rare diseases and orphan drugs, with presence in 37 mostly European and Arab countries, that aims to contribute to the improvement of the diagnosis, care, and treatment of patients with rare diseases. This approach is already adopted in the Israeli and Greek genetics communities, mostly to share genetic laboratory information, but also to accelerate the data collection process. Partnering with local human genetic societies is also liable to have the same result.

Moreover, to increase awareness among interested parties, the resulting NEMDBs should be made known to their potential users using various communication channels. These do not necessarily include international scientific journals to which local institutes in these countries might not have access, but local scientific journals, news bulletins, scientific events (symposia, workshops), one-to-one communication, and so on. Such an approach was followed by the Israeli NEMDB, by producing a review article in the Israeli Medical Association Journal, a leading peer-reviewed scientific medical journal in Israel [Zlotogora et al., 2009].

### 2. Create International Networks from Closely Related Populations to Enhance Communication, Interaction, and Initiate Research Networks Between Them

In this case, it would be mutually beneficial for closely related countries such as in Latin America, the Middle East, or regions in Africa, to initiate new or strengthen existing collaborative ties toward comprehensive genetic variation data capture. Similarly, specific resources would be committed in different countries so that there will be no redundancy in their research efforts. In this regard, the Catalogue for Transmission Genetics in Arabs (CTGA) ([Tadmouri et al., 2006]; see also below) initiated from the Centre for Arab Genomic Studies (http://www.cags.org.ae) in Dubai, United Arab Emirates, is a pilot project to document genetic disorders in Arab populations from the published literature. The current version of CTGA database contains nearly 760 full-text records for nearly 1,290 Mendelian disorders and related genes, including extensive data from the United Arab Emirates, Bahrain, Oman, and Qatar.

### 3. Foster Communication, Interaction, and Research Networks Between Developing and Developed Countries

This would be particularly beneficial in both data collection from the literature or generation of new genetic variation data and would include:

- Education of healthcare providers, the public, and government officials from researchers and health care providers from developed countries, regarding data collection, data generation and interpretation, and so on. To this end, the Pan-Arab Human Genetics conferences, with international participation from developed countries, could very well be an example of such interactions. The African Society for Human Genetics is also spearheading efforts to create a database of genetic variation in indigenous African populations (Raj Ramesar; personal communication; Al Aama and coworkers, submitted).
- Assistance in data handling and storage in large databases and servers in developed countries, particularly where massive storage units (e.g., GRIDs) are needed to accommodate large datasets. Such possibilities are currently offered by LSDB and NEMDB software developers (LOVD [Fokkema et al., 2005] and ETHNOS [van Baal et al., 2010], respectively) in The Netherlands and Greece, respectively, to either download for use or to store in their local servers, data from interested parties and curators that do not have either the knowledge or the resources needed, although ultimately the aim would be to provide the infrastructure locally.
- Consultancy in the design and coordination of large-scale genome projects (see also below) if such need arises, including patients recruitment, experimental design, software/database development (see also Point 5).
- Outsourcing of high-throughput experiments, such as microarray or next-generation sequencing experiments, to laboratories in the developed countries, where such infrastructure and expertise is more likely to be available, but also provide expert external training to those countries where the facilities are available but outstrip the local expertise.
- To work interactively, leading from all of the above, to train postgraduate students and other researchers toward capacity development in developing countries.

### 4. Ensure That the Database Management Systems That Are Being Used or Developed Can Be Utilized by Those in a Limited Resource Environment

Today, LSDBs and NEMDBs, which capture the core information in any one of these domains of information, are developed by experts and curated with invaluable skill and experience. Often, developing countries suffer from a limited resource research environment; hence, this reality should be carefully considered when database management systems are developed for this purpose.

Therefore, the database management system should be designed and built such that the resulting database would:

- Record the specific variants of inherited disorders found in a population or ethnic group in a comprehensive and user-friendly manner, that is, by providing consistent and simplified information accompanied by numerous links to other databases and related resources [Patrinos, 2006].
- Provide the end user with an interface for easy data query and retrieval of information pertaining to the mutation spectrum of each disorder together with the corresponding mutation frequencies and references.
- Allow permanent data storage and secure data entry and modification. In this case, software pipelines should also allow automated genotype data transfer between the clinic and/or laboratory to the database, and ideally from the genotyping equipment (sequencer, scanner, etc.) directly to the database.
- Operate under strict quality assurance guidelines, such as intellectual property rights, the professional code of ethics (http://ihealthcoalition.org/ethics/ehcode.html), or specific ethical guidelines [Povey et al., 2010], laws on computing and liberties (e.g., http://www.ama-assn.org/ama/pub/category/1905.html), electronic data protection, specific guidelines, and any law or regulation applicable in addition to the local ethical issues that may be specific to a given country.

Such databases should be able to operate remotely, should the required support not be available locally (see also Point 3).

### 5. Provide Support to Developing Countries to Build Capacity and to Fully Participate in the Collection, Analysis, and Sharing of Genetic Variation Information

Comprehensive genetic variation data capture usually entails highly specialized researchers or diagnostic laboratory staff and state-of-the-art infrastructure, particularly where generation of new genomic data comes to play. Developing countries usually suffer from brain drain and lack of highly sophisticated equipment to perform cutting-edge research. To this end, developed countries have the obligation to alleviate this burden and to assist developing nations by every means possible [Kaput et al., 2009]. It should be noted that developing countries have a valuable resource of clinical samples, often with very unusual phenotypes, particularly when consanguineous families are involved because in this case, the elucidation of the genetic basis of the underlying phenotype becomes much easier. Developing countries should therefore:

- Educate healthcare providers and government officials to demonstrate the need to initiate documentation of the genetic variation in their countries and to underline the benefits of cooperation in biomedical research [Bhan et al., 2007].
- Increase awareness in the general population about the need to comprehensively record the underlying genetic heterogeneity in their country and informing them objectively about the benefits and risks of such projects.
- Ascertain all parties involved that the research outcome will not be misused. Certain populations or ethnic groups in developing countries usually mistrust research involving genetic analyses or fear that results can stigmatize them or worse (http://www.eubios.info/ASIAE/BIAE201.htm). For example, Malay-Muslims, Chinese, and Indians in Singapore expressed anxiety about breach of confidentiality, the misuse of their DNA for cloning, and possibilities of being diagnosed with disease [Wong et al., 2004]. Community-based research collaborations may provide fora for addressing cultural and ethical concerns of biomedical research [Povey et al., 2010]. Analyzing the extent of human genome variation creates an ideal opportunity for the developed and the developing nations of the world to forge meaningful partnerships and to work together in an unprecedented way, initially to identify variation causing disease, and then to simplify mutation detection in specific ethnic groups, and then to understand how genetic variation contributes to human phenotypic diversity. By ensuring that all nations and ethnic groups have an equal and fair opportunity to share data and technology, evidence-based information will be provided such that all populations can benefit from a global society health network.
- Emphasize in every stage of the collaborative project that external input is needed from the design and coordination of NEMDB development and curation to database hosting and technical support, patient recruitment, and experimental design to performing high-throughput experiments in the case of large-scale genome projects (see also Point 3). To this end, funding agencies (e.g., UKIERI; UK-India Education and Research Initiative) could assist by funding innovative projects so that developing countries become independent and self-sustained.
- Develop partnerships for the equitable sharing of knowledge and technology between developed and developing countries. These partnerships should be ideally formed under the umbrella of international organizations, such as the HGVS or the Human Genome Organization (HUGO; http://www.hugo-international.org) that would also organize interinstitutional specialized educational programmes and workshops. The biennial mutation detection workshop would be a paradigm for this initiative and may sometimes be held in developing countries.

### 6. Develop a Framework to Facilitate Interactions Between the Coordinating Center and National, Regional, and International Agencies

Researchers and their academic institutions that participate in national data collection projects and/or NEMDBs development should jointly develop infrastructures that would ensure smooth communication between them and national and international agencies. These include: (1) national governments and their Departments of Health, Science and Technology, Development, Education and so on, local human genetic societies; (2) regional organizations, such as the NEPAD (New Programme for Africa's Development); and (3) international organizations, such as the European Commission (EC), United Nations (UN), United Nations Children's Fund (UNICEF), World's Health Organization (WHO), Organization for Economic Cooperation and Development (OECD), etc.

This, in turn, would: (1) create new collaboration ties with neighboring countries interested in initiating similar projects, (2) increase the chances of receiving funding for these activities (see also below), and (3) increase visibility of the progress to the international scientific community.

### 7. Ensure That All Ethical, Legal, Religious, and Social Issues Are Thoroughly Considered when NEMDBs and/or Data Capture Projects Are Launched in Developing Countries

The goal of establishing LSDBs and NEMDBs in developing countries is the sharing of genetic variation information for the benefit of the respective populations. This includes the protection of privacy, which in this context is the right of the individual and members of their family to be protected against intrusion into their personal information and further intrusions ensuing from access to this, by publication of information. Of course, there is no such issue when summary level data are reported for a population, as it is the case in some NEMDBs. Therefore, there should be a balance between the public's interest in the value of the shared information and its interest in the strict protection of privacy. This balance would be viewed differently in different cultures and ethnicities [Al-Aqeel, 2007].

For many of these NEMDBs, it seems reasonable that an independent group of qualified scientists oversees these resources and related activities not only at their initiation but on an ongoing basis. Where needed, the main purpose of the particular NEMDB should be clarified, recognizing that this may change with time. This will allow evaluation of the exact requirements for data deposition in the NEMDBs, and whether compliance with the remaining guidelines will be possible with the database content freely available to the public or partly restricted to registered persons, namely curators, physicians, and so on. This measure was implemented in the Israeli NEMDB, where not all information is made publicly available [Zlogotora et al., 2007], because access to specific cases is password-protected [Zlotogora et al., 2009]. A similar measure would entail anonymizing genetic data, either by permanently removing or coding the personal identifiers [Povey et al., 2010]. Of interest, the newest version of the LOVD software [Fokkema et al., 2005] has the option to store data that are not public but that can be queried. The result of a query hitting nonpublic data is a notification that there is such information in the database but that the curator needs to be contacted to get more information.

The depth of the ethnic and geographic origin data should be determined in advance, particularly in the case of small villages where carriers of rare variants could be identified even if personal information were not provided. In this case, data donors should be well informed and provided with an explanation as to why their data documentation is useful and how their privacy is secured. These individuals should consent to the inclusion of their genetic information in the database and reserve the right to withdraw their data from the database whenever they desire. This is particularly important for persons unable to consent because of disability or young age. Standardized consent forms need to be translated to different local languages and/or dialects.

Finally, these databases would also benefit from some independent/external ethical review, perhaps from an international group (HGVS or HVP) who could advise them accordingly.

## Nation-Wide Genomic Projects that Would Serve as Paradigm for Genetic Variation Data Collection

By definition, a nation-wide genomic project is a national multicentre initiative to determine the underlying genetic heterogeneity of a population with the goal to ascertain the genetic variation leading to common or rare inherited diseases. Such initiatives consist of three parts: (1) documenting the genetic heterogeneity that has already been reported in the published literature, (2) generating new genetic data by screening a large proportion of the population using high throughput genotyping approaches, and (3) accumulating data causing inherited disease from hospitals, laboratories, and other healthcare facilities. In every case, except perhaps (3), a large national genetic database/portal is being developed to accommodate the sheer amount of data gathered. To date, there are few nation-wide genomic projects in various developing countries that are currently in progress. These projects (summarized in Table 1) implement some of the guidelines described previously. Also, there are several other NEMDB projects, all of which more or less follow these guidelines, that have already been reviewed [Patrinos, 2006; van Baal et al., 2007] and will, therefore, not be further discussed because they lie outside the scope of the present article.

**Table 1:** Nation-wide Genome and Related Projects

Project	URL	Aims and scope	References
*Nation-wide genome projects*			
The Estonian Genome Project	http://www.geenivaramu.ee/index.php?lang = eng	To create a database of health, genealogical, and genome data representing approximately 10% of Estonia's population	Metspalu, 2002
The MEDGENET Project	http://medgenet.tredueuno.it	To expand the human expertise in clinical genetics in Mediterranean Partner Countries (MPC) that share a common burden of genetic diseases.	van Baal et al., 2010
The Indian Genome Variation database	http://www.igvdb.res.in	To create a DNA variation database of the people of India and make it available to researchers for understanding human biology with respect to disease predisposition, adverse drug reaction, population migration	Indian Genome Variation Consortium, 2005
The Catalogue of Transmission Genetics in Arabs	http://www.cags.org.ae/ctga_search.html	To provide the user with clinical and molecular genetic descriptions of genetic disorders, epidemiological details of reports of the disorders in the Arab population and clinical case details wherever available	Tadmouri et al., 2006
*Other related projects*			
Kuwaiti Molecular Genetics Diagnostic Service	http://www.al-mulla.org	To provide autozygosity mapping services, Information on Arab genome and molecular karyotying of cancers	N/A
UK diagnostic mutation database	http://ngrl.org.uk/Manchester/dmudb.html	To provide a route for sharing mutation data within and between diagnostic laboratories in the United Kingdom to support genetic testing services for patients.	N/A
Hellenic Genome Variation database	http://www.goldenhelix.org/hellenic	To create a compendium for genetic diseases and to encourage genetic studies to elucidate the genetic basis of inherited diseases in Greece	Patrinos et al., 2005
The International Society for Gastrointestinal Hereditary Tumours (InSiGHT)	http://www.insight-group.org	To improve the quality of care of patients and their families with any condition resulting in hereditary gastrointestinal tumours	N/A
International Thalassemia Network (ITHANET)	http://www.ithanet.eu	To strengthen research and treatment of hemoglobinopathies	Lederer et al., 2009
Pharmacogenomics for Every Nation Initiative	http://www.pgeni.org	To determine the frequencies of the commonest pharmacogenomic markers in various populations, particularly from developing countries worldwide	N/A

N/A, not available.

The Estonian Genome Project (EGP; http://www.geenivaramu.ee/index.php?lang = eng) is a research venture of the University of Tartu initiated in 2001 [Metspalu, 2002] and aims to create a database of health, genealogical, and genetic data representing approximately 10% of Estonia's population. The database will make it possible for researchers both in Estonia and outside to look for links between genes, environmental factors, and common diseases (such as cancer, diabetes, depression, cardiovascular diseases, etc.). Currently, EGP gene bank contains data contributed by over 20,000 donors with an expected increase to 100,000 within the next 3 or 4 years.

The Indian Genome Variation database (IGVdb; http://www.igvdb.res.in) is a consortium activity established in 2003 to document the inherent genetic variability of the Indian subpopulations as a first step toward identifying genetic biomarkers to understand susceptibility for any genetic disease and population migration, pharmacogenomic markers to understand variable drug response. The Indian Genome Variation (IGV) consortium aims to provide data on validated SNPs and repeats, both novel and reported, along with gene duplications, in over 1,000 genes (selected on the basis of their relevance as functional and positional candidates in many common diseases including genes relevant to pharmacogenomics), in 15,000 individuals drawn from Indian subpopulations [Indian Genome Variation Consortium, 2005].

Kuwait established within the Faculty of Medicine, Department of Pathology a Molecular Genetics Diagnostic Service Division (http://www.al-mulla.org), integrated within a research laboratory, which is focused on delivering state-of-the-art diagnostic service for the Kuwaiti population. These services, which focus on autozygosity mapping in families with consanguineous marriages, provide the research laboratory with a unique opportunity to identify novel genes in autosomal recessive disorders. Moreover, various unique cases suffering from cancer are currently being analyzed using the Affymetrix Array 6.0 microarray technology to identify copy number changes and homozygosity stretches. The data generated from these studies are deposited in CAGS database (see above), and LOVD, whereas the Kuwaiti NEMDB has been recently established databases to encourage data storage for genetic diseases in different subpopulations in Kuwait.

The United Kingdom has developed a central database to collect all UK genome variation data, and their effects, from participating diagnostic laboratories. The diagnostic mutation database (DMuDB; http://ngrl.org.uk/Manchester/dmudb.html) provides a route for sharing mutation data within and between diagnostic laboratories in the United Kingdom to support genetic testing services for patients. Currently DMuDB has over 4,884 records for 33 genes, namely Breast Cancer (*BRCA1, BRCA2*), Lowe Syndrome (*OCRL*), Sotos Syndrome (*NSD1*), HNPCC (*MLH1, MSH2*), cystic fibrosis (*CFTR*), X-linked retinitis pigmentosa (*XLRP*), neurofibromatosis, Types I and II (*NF1, NF2*), Alstrom Syndrome (*ALMS1*), CADASIL (*NOTCH3*), familial adenomatous polyposis (*FAP*), and muscular dystrophy (*DMD*), containing over 10,985 individual variants.

The Hellenic Genome Variation database aims to document the genetic heterogeneity in the Hellenic population. The first version of the Hellenic NEMDB was created in 2004 [Patrinos et al., 2005]. In 2007, research institutes involved in hemoglobinopathies research initiated a pilot multicenter project to determine the mutation spectrum of the thalassemia syndromes in various geographical regions in the country with the goal to document the findings in the Hellenic NEMDB. These data showed that although Greece is a rather homogeneous country, there are big qualitative (type of mutations) and quantitative (allelic frequencies) differences in various parts of the country, namely, central Greece [Samara et al., 2007] and South-Western Greece [Papachatzopoulou et al., 2010]. These data compared to findings from North-Western Greece [Georgiou et al., 2003] and the rest of the country [Patrinos et al., 2005] reveal valuable information for region-specific population carrier screening and for efficient provision of prenatal diagnosis of β-thalassemia in Greece.

Finally, there are four international consortia with activities related to the documentation of causative mutations and allelic frequencies in developing countries:

- The International Society for Gastrointestinal Hereditary Tumours (InSiGHT; http://www.insight-group.org) is an international multidisciplinary, scientific organization with a mission “... to improve the quality of care of patients and their families with any condition resulting in hereditary gastrointestinal tumours.” The InSiGHT consortium maintains a LOVD-based database for genetic variations for the *EPCAM*, *MLH1*, *MLH3*, *MSH2*, *MSH6*, *MUTYH*, *PMS1*, and *PMS2* genes leading to gastrointestinal tumors and encourages the establishment of national repositories for patients suffering from gastrointestinal tumors, particularly in developing countries.
- The International Thalassemia Network (ITHANET; http://www.ithanet.eu) is a European Commission-funded project that comprises a network of all major European research institutions active in the field of molecular and clinical research of thalassemia and related hemoglobinopathies [Lederer et al., 2009]. Among its activities, ITHANET hosts a database (ITHANETbase) that includes mutation frequencies in various populations at-risk for thalassemia, particularly from developing countries. In many instances though, this repository is largely redundant to HbVar database of hemoglobin variants and thalassemia mutations (http://globin/bx.psu.edu/hbvar) [Patrinos et al., 2004].
- The MEDGENET project (http://medgenet.tredueuno.it) is a European Consortium, funded by the European Commission, aiming to expand the human expertise in clinical genetics in Mediterranean Partner Countries (MPC) through the transfer of knowledge and technology between the two rims of the Mediterranean that share a common burden of genetic diseases. Among the actions foreseen by MEDGENET is the building of Medical Genetics databases for the various partner countries. Several NEMDBs are built from scratch, such as the Tunisian and Egyptian NEMDBs or expanded from the existing NEMDBs, such as the Israeli [Zlotogora et al., 2007], Cypriot [Kleanthous et al., 2006], and Lebanese NEMDBs [Mégarbané et al., 2006], using the ETHNOS database management system for NEMDB development and curation [van Baal et al., 2010].
- The Pharmacogenomics for Every Nation Initiative (PGENI; http://www.pgeni.org) that aims to determine the frequencies of the commonest pharmacogenomic markers in various populations, particularly from developing countries worldwide.

Finally, there are a few nation-wide DNA biobanks, such as the National Laboratory for the Genetics of Israeli Populations, representing a national repository for human cell lines and DNA from the unique and large ethnic variation of the Israeli populations (Tel Aviv University; available at http://www.tau.ac.il/medicine/NLGIP) or the Biomaterial Banking Medical Research Initative (BBMRI) in Malta, which of course lie at the borderline of the nation-wide genomic projects described above.

## Funding and Incentives

There are currently two main obstacles that hinder further efforts to expand the NEMDB field, namely, funding and providing incentives to data submitters.

Funding for collecting genetic variation data has traditionally been difficult due to the extreme fragmentation of the field [Patrinos and Brookes, 2005]. Therefore, it would be expected that efforts to receive funding for genetic variation data capture are more likely to flourish if treated as concerted efforts. GEN2PHEN (http://www.gen2phen.org) represents such initiative that have already received funding from the EC and aims to unify human and model organism genetic variation databases toward increasingly holistic views into genotype-to-phenotype data, and to link this system into other biomedical knowledge sources via genome browser functionality. Linking population and ethnic-specific information into LSDBs and diagnostic databases, creating NEMDBs and developing genomic databases, similar to the Indian Genome Variation database are key activities for the main GEN2PHEN work packages. Obviously, other strategically minded funding agencies (e.g., NIH), charities, and foundations would also launch similar calls. Recently the Australian Node of the HVP has been funded to pilot software for data flow from hospitals/clinics via country specific databases to LSDBs and NCBI/EBI/UCSC browsers (unpublished). Involving universal organizations such as the WHO and UNESCO in the fund-raising project will clearly have a strong impact.

On the other hand, the development of comprehensive analyses of human genetic variation often reaches a barrier in the form of the reward system for clinicians and academic researchers. First of all, database entries could be made a mandatory quality control standard for clinical laboratories and clinicians. Notably, according to a new initiative of the Israeli government, genetic laboratories are mandated to report the details of the tests they are performing in the Israeli NEMDB in order to obtain accreditation from the Israeli Ministry of Health [van Baal et al., 2010].

For researchers, a publication or Web-based system establishing microattribution and community annotation of mutations and cited data will enable measurable contributions to the scientific knowledge base [Anonymous, 2008; Hoffman, 2008]. Similarly, database journals would also serve this task by providing a forum for publishing gene variation data that would be eventually deposited in the PubMed literature database. One such journal, the open access Human Genomics and Proteomics (http://www.sage-hindawi.com/journals/hgp), which is affiliated with FINDbase (http://www.findbase.org) [van Baal et al., 2007], will focus on studies characterizing causative mutation and/or biomarker frequency spectra. Accepted contributions including datasets will be linked in FINDbase and deposited in PubMed [Patrinos and Petricoin, 2009]. Also, the journal *Human Genetics* has been accepting mutation data and this is linked to HGMD (http://www.hgmd.org).

Journals, tenure, and promotion committees, and funding agencies would be encouraged to cite these contributions to and citations of LSDB and international national databases [Patrinos and Brookes, 2005]. The rapid progress in genetic research has led to a declining interest of scientific clinical genetic journals in case reports with single or few novel mutations. This may deter clinicians from reporting these new mutations, which collectively become important, unless another venue becomes visible.

## Concluding Remarks

As stated above, documenting population/ethnic-specific data in developing countries are among the primary objectives of the HVP that appreciates the genomic sovereignty/equality for all countries to be involved and acknowledges the value of “human capital” within all populations. Real and tangible benefits of the HVP to improve health will be generated for participating populations. Hence, the voluntary participation of the greatest number of countries would ensure a more general applicability, and it is hoped that many countries will eventually decide to participate.
